# Facile Preparation of Charcoal Nanomaterial from Fishery Waste with Remarkable Adsorption Ability

**DOI:** 10.3390/ma12081318

**Published:** 2019-04-23

**Authors:** Yaning Wang, Yarui Zhou, Lu Cai, Jian Guo, Yong Xu, Hailong Zhang, Lili Ji, Wendong Song

**Affiliations:** 1 Institute of Innovation & Application, Zhejiang Ocean University, Zhoushan 316022, China; wynzjou@126.com; 2 School of Naval Architecture and Mechanical-Electrical Engineering, Zhejiang Ocean University, Zhoushan 316022, China; zyr0612Z@gmail.com; 3 College of Environmental and Science Technology, Donghua University, Shanghai 201620, China; cailu@zjou.edu.cn; 4 College of Food and Medical, Zhejiang Ocean University, Zhoushan 316022, China; guojian@zjou.edu.cn; 5 Zhoushan National Oil Reserve Base Co., Ltd., Zhoushan 316022, China; xuyong@sinochem.com; 6 College of Petrochemical and Energy Engineering, Zhejiang Ocean University, Zhoushan 316022, China; swd60@163.com

**Keywords:** fishbone charcoal, modification, adsorption capacity, removal efficiency

## Abstract

In this study, modified activated fishbone charcoal (MAFC) was successfully prepared to remove emulsified oil from oily wastewater. Various characteristic techniques, including SEM, XRD, FTIR, and BET, were employed to investigate the morphology, texture, and surface properties of as-prepared samples. BET results demonstrated that the specific surface area of fishbone charcoal increased from 69.8 m^2^/g to 206.0 m^2^/g after treatment with K_2_CO_3_ as an activating agent, while the total pore volume of MAFC increased from 0.003 cm^3^/g to 0.3 cm^3^/g, accompanied by the formation of abundant pore structures. It was observed that 90.1% of emulsified oil (100 mg/L) was successfully removed by MAFC under our experimental conditions. The results of a kinetic and isotherm model analysis indicated that the adsorption experimental data were not only consistent with the Langmuir adsorption isotherm but were also well-described by the pseudo-second-order adsorption model. It is expected that this highly efficient and inexpensive MAFC can be a promising bio-adsorbent for removing organic pollutants from industrial wastewater.

## 1. Introduction

Oily wastewater can impact on the environment in negative and harmful ways and may result in serious environmental pollution. There are many sources of oily wastewater, including accidental marine oil spill, wastewater from oil exploitation and oil tank cleaning, and ballast water. The direct discharge of oily wastewater will severely threaten human health and lead to long-term ecological environmental distress [1,2]. There are many technologies available for the treatment of oily wastewater, which can be divided into three categories: mechanical, chemical, and biological treatment methods [3,4,5,6,7]. Nevertheless, these techniques have several limitations, such as secondary pollution to the environment, a complicated operation process, high costs, and high time consumption. Traditionally, adsorption has been the preferred method for researchers due to its emulsification degree and its size of oil droplets in oily wastewater [8,9].

Porous biochar materials are a promising solution because of their large surface area, abundant pore structure, the hydrophobic nature of their surface, and their mechanical and thermal stability [10,11,12,13]. In recent decades, it has been noted worldwide that biochar materials can be utilized as an adsorbent. Jian and co-workers have studied the adsorption property of biochars derived from rice husk, which has a high adsorption capacity for methylene blue, iodine, and copper ions from aqueous solution, due to its ion-exchange and complexation properties [14]. Various biochars pyrolyzed at 700 °C were obtained for the removal of organic contaminants. Correlations between the adsorption behavior and the physicochemical properties of biochars were studied and analyzed, while the mechanism and driving forces responsible for adsorption were also explored [15]. Research on adsorption kinetics, isotherms, and thermodynamics, and the mechanism of adsorption of methylene blue (MB) has been conducted on biochar prepared from the co-pyrolysis of municipal sewage sludge and tea waste [16]. Results indicated that the interaction between MB and biochar involved electrostatic interaction, ion exchange, surface complexation, and physical interaction. A novel biochar–AlOOH nanocomposite with excellent adsorption capacities for various contaminants in aqueous solutions has also been developed [17]. Moreover, compared with other processing methods, the biochar adsorption method has many advantages, such as a small occupied area, high adsorption efficiency, easy operation, and good effluent quality, which can be effectively used for the advanced treatment of oily wastewater [18,19]. By adding biochar materials into emulsified oily wastewater, the experimental system will successfully realize a change from liquid to semi-solid, leading to an easier removal of emulsified oil from oily wastewater [20,21,22,23]. In particular, biomass wastes have become potential raw materials for the production of carbon nanostructures due to their abundant availability and environmental friendliness [13,19]. Waste fishbone is predominantly composed of inorganic compounds with a small number of organic compounds [24]. It is well known that hydroxyapatite (Ca_10_(PO_4_)_6_(OH)_2_, HAp) is the main component in inorganic porous substances and has outstanding adsorption properties by complexing with phosphate and hydroxyl groups [25,26]. For instance, Wang et al. investigated the adsorption performance of fishbone charcoal derived from grass carp fish on Pb (II) ion [27]. Ehab et al. suggested that fishbone biochar extracted from Mullet fish has a high adsorption capacity for the removal of hazardous heavy metals from wastewater [28]. Natural fishbone apatite has been prepared and can achieve its highest Pb removal rate of 24.76% when the ratio of fishbone to fly ash is 20%, after a 72 h leaching process [29]. These studies have demonstrated that fishbone material may be an excellent and low-cost adsorbent for the removal of environmental pollutants. However, to our best knowledge, the utilization of fishbone for the preparation of oily wastewater adsorbent has not been reported elsewhere.

Chemical activation has been widely used as an effective method for producing excellent biochar materials with high surface areas and abundant pore structures. In general, the mechanism of chemical activation includes hydroxide reduction and carbon oxidation [30]. Other commonly used chemical reagents, such as ZnCl_2_, KOH, and NaOH, are hazardous, corrosive, and environmentally unfriendly, causing secondary pollution to the ecosystem [31,32,33,34]. Therefore, as a harmless, mild, and benign chemical substance, K_2_CO_3_ has gained many application prospects.

The main objective of this paper was to develop a novel fishbone charcoal nanomaterial and to assess its adsorption performance for removing emulsified oil from oily wastewater. In this study, we demonstrated the facile preparation of modified activated fishbone charcoal (MAFC) via a combination of pyrolysis and modification by K_2_CO_3_ impregnation. The effectiveness of this modified adsorbent for removing emulsified oil was investigated under various environmental conditions, with different adsorbent doses, contact times, contact temperatures, and initial oil concentrations. In addition, the structure, morphology, and surface properties of the synthesized samples were characterized by XRD, SEM, and FTIR. The unique features of MAFC indicated its great potential in the engineering field for environmental remediation.

## 2. Materials and Methods

### 2.1. Materials

Waste fishbones were collected from a local food market in Zhoushan, Zhejiang. We purchased 0# diesel oil from Zhoushan Petrochemical Co., Ltd., (Zhoushan, China). All the other inorganic and organic chemicals were of analytical grade and were obtained from Sinopharm Chemical Reagent Co., Ltd., Shanghai, China. All reagents used in the study were of analytical grade.

### 2.2. Preparation of Fish Charcoal Material

The collected fishbones were thoroughly washed several times with 100 °C distilled water, followed by drying at 120 °C for 12 h in an oven. Then, 10 g dried fishbone was pre-carbonized at 750 °C for 2 h in nitrogen (99.995%) flow (100 cm^3^/min), with a temperature ramp of 10 °C/min. The carbonized fishbone (FBC) was allowed to cool to room temperature under N_2_ gas flow.

During activation, the resulting char was stirred in K_2_CO_3_ solution (the ratio of carbon to K_2_CO_3_ was 1:3, wt/wt %) for 24 h and then activated in a vacuum tube furnace at 750 °C for 90 min. The same temperature ramp and nitrogen flow were adopted for the pyrolysis process and activation experiment. The K_2_CO_3_-modified fishbone (MAFC) was washed with de-ionized water until the pH was adjusted to 7.0–7.2 and was then dried at 110 °C in an oven for 3 h, before being used in the following experiments.

### 2.3. Characterization

The composition and morphology of the samples were investigated by scanning electron microscopy (SEM, Hitachi S-4800, Tokyo, Japan) and X-ray diffraction (XRD, Ultima IV X-Ray Diffractometer, Rigaku Corporation, Tokyo, Japan) in the range of 2θ = 10°–80°. The surface functional groups before and after activation were analyzed by Fourier transform infrared spectra (FTIR, Nicolet 5700, Thermo Corp., Waltham, MA, USA). The specific surface areas of the samples were measured with a Micromeritics ASAP 2010 instrument and analyzed by the BET method.

### 2.4. Adsorption Experiment

Sorption performance of MAFC was investigated by testing the removal efficiency of emulsified oil from oily wastewater. The experimental system was synthesized by mixing 0# diesel oil and distillated water for 30 min using ultrasound. Adsorption experiments were carried out in 100 mL Erlenmeyer flasks containing the initial oil concentration (50 mL), which ranged from 30 mg/L to 300 mg/L (100 mg/L for the adsorption kinetics study), then, 0.1 g MAFC was added into the flasks with an initial pH of 7. The mixtures were continuously shaken at 120 rpm in a constant temperature oscillator for 30 min. The residual oil concentration in the supernatant was analyzed by measuring light absorbance at a wavelength of 255 nm using an UV–Vis (UV-2250 Shimadzu, Tokyo, Japan) spectrometer [35]. Two possible key factors, adsorbent dose and reaction temperature, were investigated in several batch experiments.

The adsorption property of the samples was determined by using the following equation:

Adsorption capacity (mg/g) = (C_0_ − C_e_) × (V/m) (1)

where q_e_ is the equilibrium adsorption capacity of samples, mg/g, C_0_ (mg/L) is the initial concentration of the emulsified oil, C_e_ (mg/L) is the residual oil concentration in the supernatant, V is the volume of the solution, L, and m is the mass of the adsorbent, g.

Adsorption = [(A_0_ − A_1_)/A_0_] × 100% (2)

where R is the removal percentage of emulsified oil, A_0_ is the initial absorbance of emulsified oil, and A_1_ is the absorbance measured at a definite time.

### 2.5. Recyclability Experiments

Recyclability experiments were implemented to evaluate the efficiency of an as-prepared adsorbent. First, 0.1 g of MAFC was added to 50 mL of emulsified oil solution (100 mg/L) for 30 min of adsorption. Following centrifugation the MAFC was placed in an Erlenmeyer flask, and 25 mL of n-hexane was added. This was followed by the process of desorption, for 30 minutes. At the end, the sample was dried in an oven at 60 °C for 12 h.

### 2.6. Main Kinetics Models

Main kinetics models were used to investigate the emulsified oil adsorption kinetics on the raw fishbone (RFB), FBC, and MAFC.

The adsorption kinetics modes were denoted by the following equation:

- Pseudo-first-order model
  Ln(q_e_ − q_t_) = lnq_e_ − k_1_t (3)
  where q_e_ and q_t_ are, respectively, the amounts of emulsified oil (mg/g) adsorbed at equilibrium and at time t. K_1_ is the rate constant of the pseudo-first-order (min^−1^).
- Pseudo-second-order model
  (4) $$\frac{t}{q_{t}}=\frac{1}{K_{s}q_{e}^{2}}+\frac{1}{q_{e}}t$$
  where q_e_ and q_t_ are the amounts of emulsified oil (mg/g) adsorbed at equilibrium and at time t, respectively. K_s_ is the rate constant of the pseudo-second-order (min^−1^).

### 2.7. Adsorption Isotherms Models

Thewo isotherm models used were denoted by the following equation:

- Langmuir model
  (5) $$\frac{C_{e}^{\prime }}{q_{e}^{\prime }}=\frac{1}{q_{\max}b}+\frac{1}{q_{\max}}C_{e}^{\prime }$$
- Freundlich model
  (6) $${\mathrm{lnq}}_{e}^{\prime }={\mathrm{lnK}}_{f}+\frac{1}{n}{\mathrm{lnC}}_{e}^{\prime }$$
  where C_e_ (mg/L) is the equilibrium concentration in the solution, q_e_ (mg/g) is the emulsified oil adsorbed at equilibrium, b (mg/g) is the maximum adsorption capacity, n is the Freundlich constant related to adsorption intensity, and q_max_ (mg/g) and K_f_ ((mg/g) (L/mg)^1/n^) are the adsorption constants for Langmuir and Freundlich models, respectively.

## 3. Results and Discussion

### 3.1. Characterization of Fishbone Nanomaterials

**Figure 1:**
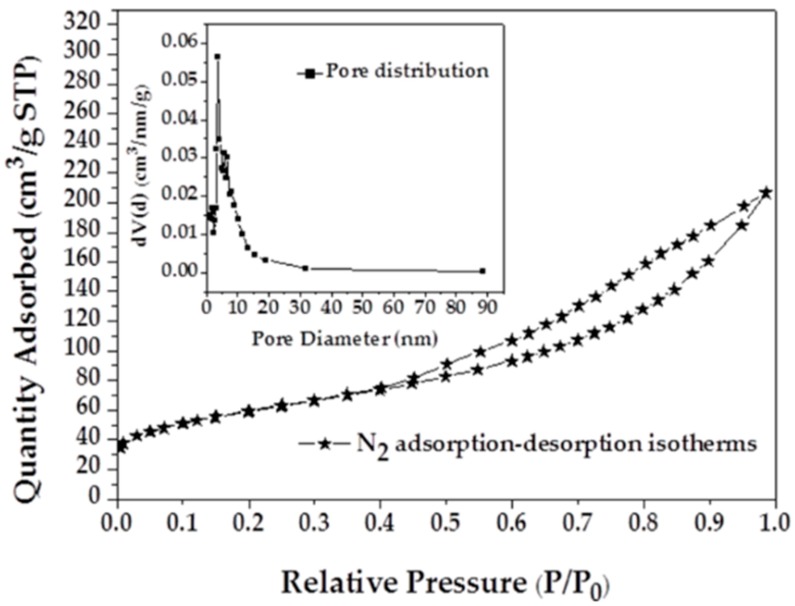
Nitrogen adsorption isotherms of modified activated fishbone charcoal (MAFC) with corresponding pore size distribution.

Nitrogen adsorption isotherms and the pore size distribution (PSD) of MAFC are shown in Figure 1. It can be observed that the nitrogen adsorption isotherms of MAFC fit well with type IV isotherms, indicating the existence of both micropores and mesopores. The adsorption capacity of nitrogen increases rapidly with the increase of relative pressure after p/p_0_ > 0.4, which suggests the existence of mesoporous structures. The pore size distribution of MAFC also exhibits a significant presence of mesopores, with pores mainly in the range 2–20 nm. Specific surface area parameters of fishbone materials before and after activation are listed in Table 1. PSD and BET results indicate that K_2_CO_3_ activation of fishbone can produce a well-developed and evenly distributed porous structure. The high specific surface area and porous network of MAFC is conducive to increasing its adsorption capacity.

As shown in Table 1, the pore structure was significantly improved via pyrolysis and the modification of K_2_CO_3_ impregnation. BET results of fishbone materials showed an increase from 0.7 to 206.0 m^2^/g, indicating abundant active sites, and an increase in the binding sites for organic molecules or pollutants, i.e., a significant increase in the adsorption activity of adsorbent [36]. It is presumed that the potassium compound could promote gasification to widen the existing pore structures and create new pores during the activation process [37], according to the following reactions (7–9) [38,39]:

K_2_CO_3_ + 2C → 2K + 3CO (7)

K_2_CO_3_ → K_2_O + CO_2_ (8)

K_2_O + 2C → 2K + CO (9)

**Figure 2:**
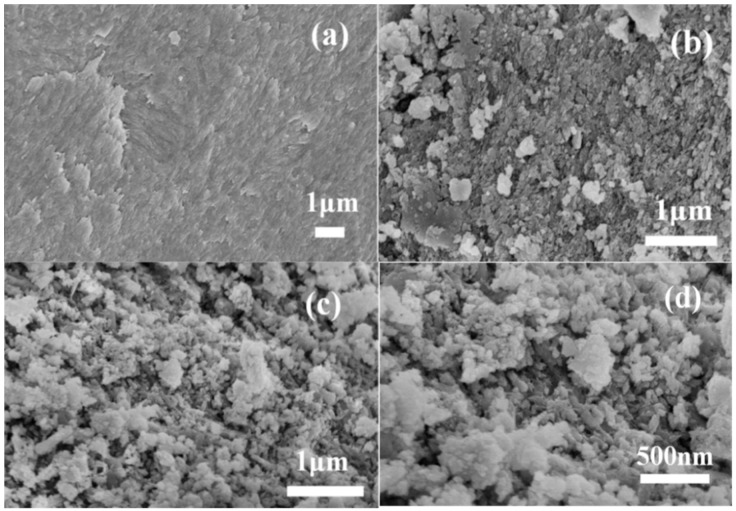
Surface morphologies of raw fishbone (RFB) (**a**), carbonized fishbone (FBC) (**b**), and MAFC with different magnifications (**c**,**d**).

Figure 2 illustrates surface morphologies of RFB, FBC, and MAFC samples visualized using SEM. It can be seen from Figure 2a that raw fishbone has a very smooth and clean surface without impurities or debris attached. In Figure 2b, FBC exhibits an uneven structure, which confirms the presence of heterogeneous porous structures, and a small amount of tiny crystal structures with an average diameter of 20–400 nm appear on the surface of FBC. After further modification, the surface of MAFC (Figure 2c,d) appeared to have a more porous structure and was much rougher than other as-prepared samples. The results demonstrated that the internal structures of MAFC were further opened and exposed after impregnation with K_2_CO_3_, which increased the number of adsorption sites in MAFC, and was beneficial to the adsorption performance of MAFC.

**Figure 3:**
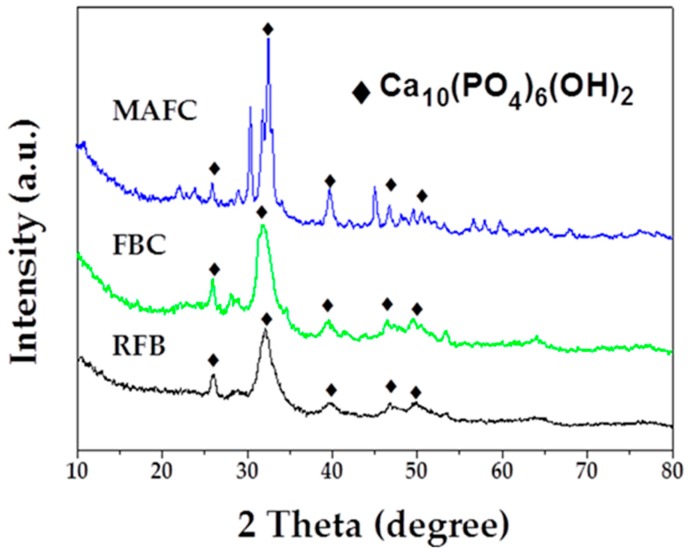
XRD patterns of RFB, FBC, and MAFC.

The crystalline structure and the purity of as-prepared samples were assessed using X-ray diffraction (XRD). RFB showed a crystalline structure with peaks at angles (2θ) of 25.9°, 32.2°, 39.8°, and 46.7°, which could be indexed as (002), (112), (310), and (222) (PDF# 09-0432). After carbonization, the data of FBC charcoal also displayed a consistent crystalline structure, with peaks of the fishbone charcoal located at angles (2θ) of 25.9°, 31.8°, 32.2°, 39.8°, and 46.7°, which could be perfectly indexed as (002), (211), (112), (310) and (222). There may have been a small amount of graphite, however, it may have been in the amorphous phase, as observed at approximately 30° for the sample FBC. After further activation, we observed that MAFC peaks located at 25.9°, 31.8°, 32.2°, 39.8°, and 46.7° could be indexed as (002), (211), (112), (310), and (222) (PDF# 09-0432). XRD analysis of hydroxyapatite [40] presents peaks at angles 2θ = 25.9°, 31.9°, 39.8°, and 46.4°. These data indicate that the main component in fishbone and fishbone charcoal was hydroxyapatite. As shown in Figure 3, the intensity of the diffraction peaks at around 2θ = 32.2° increased significantly after K_2_CO_3_ activation [23,41], suggesting that a well-developed calcium hydroxyphosphate skeleton structure had been fully constructed in MAFC. This may be confer MAFC excellent adsorption performance and the ability to restrain emulsified oil molecules.

**Figure 4:**
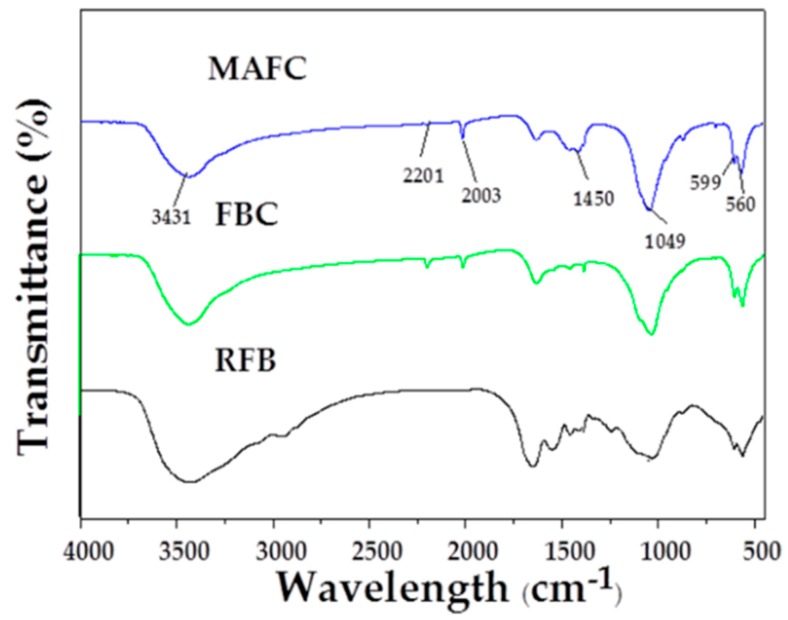
FTIR spectra of RFB, FBC, and MAFC.

The FTIR spectra of RFB, FBC, and MAFC are illustrated in Figure 4. A broad peak at around 3450 cm^−1^ is observed in RFB, FBC, and MAFC, which was assigned to O–H stretching vibration [27]. However, compared with RFB, this peak intensity was obviously reducing in both FBC and MAFC, possibly due to the loss of water and some small molecular substances. New functional groups, such as –C≡C– (2201 cm^−1^) and –C=N– (2003 cm^−1^), generated after pyrolysis and K_2_CO_3_ activation, indicating the reduction of non-polar aliphatic functional groups in fishbone biochar and the increase of the aromatization degree. Furthermore, the bands at 1450 cm^−1^ could be assigned to –CO_3_– groups. In particular, the typical characteristic peaks of MAFC at 560 cm^−1^, 599 cm^−1^, and 1025 cm^−1^ were attributed to –PO4^3−^ groups [42], consistently with data previously reported for apatite-based samples [26,43,44,45]. Therefore, MAFC was proven to have abundant unsaturated groups. The formation of π–π interactions between unsaturated groups on the surface of MAFC and aromatic rings of organic compounds, suggests a strong affinity for interaction with organic species or substances, which renders MAFC a prospect in the removal of aromatic hydrocarbons from oily wastewater.

### 3.2. Adsorption Kinetics

**Figure 5:**
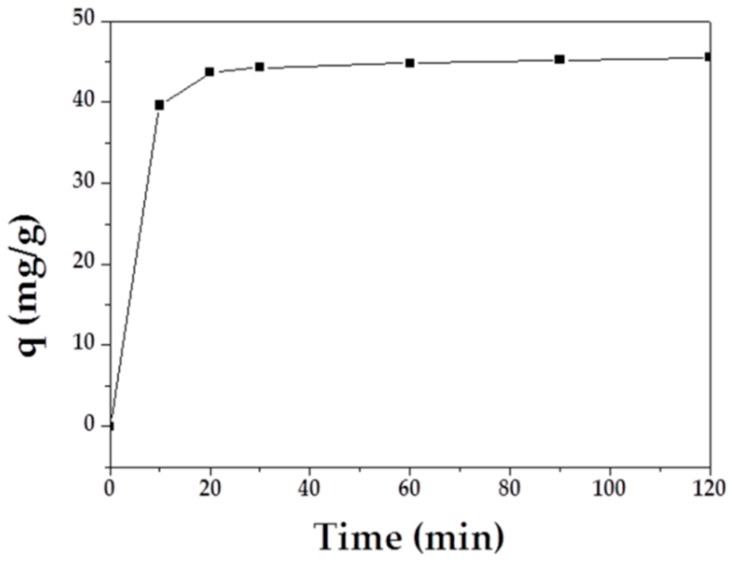
Effect of contact time on the adsorption of MAFC to emulsified oil.

The adsorption capacity of MAFC to emulsified oil over time is shown in Figure 5. The results demonstrated that emulsified oil adsorption initially increased rapidly, and the curve tended to plateau after 30 min. Therefore, it can be inferred that the adsorption equilibrium was reached after 60 min.

**Figure 6:**
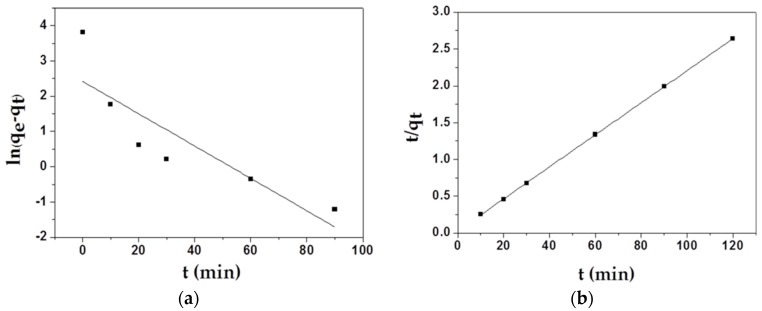
Kinetic fitting plots of the pseudo-first-order equation (**a**) for MAFC; kinetic fitting plots of the pseudo-second-order equation (**b**) for MAFC.

Two main adsorption kinetics models were employed to study the adsorption mechanism of MAFC to emulsified oil. The kinetic fitting plots of the pseudo-first-order equation for the adsorption of MAFC and the kinetic fitting plots of the pseudo-second-order equation for the adsorption of MAFC are displayed in Figure 6, and some parameters used in theoretical calculation are listed in Table 2. As shown in Table 2, the correlation coefficient of the pseudo-second-order kinetics model (R^2^ = 0.99996) was larger than that of the first-order model ((R^2^ = 0.70651), and the q_e_ value calculated from the plot (46.08 mg/g) was closer to the actual experimental value (45.5 mg/g). Therefore, all data indicated that the second-order kinetic model was suitable for describing the real adsorption process, suggesting that the sharing or exchange of electrons between the adsorbent and the adsorbate played a dominant role in the adsorption process.

The utilization of carbon material as an adsorbent has been well addressed. Ngarmkam and coworkers [46] carried out research on the removal and recovery of residual oil onto palm shell-based carbon; the equilibrium adsorption capacity of samples reached 30–90 mg/g. Cai et al. [47] explored the adsorption properties of diesel oil on modified crab shell-activated biochar carbon; the adsorption capacity was 93.9 mg/g. Although the oil sorption capacity of MAFC obtained in this experiment was 45.5 mg/g, which is relatively low compared with other adsorbents, waste fish bone materials have potential to be applied for the removal of oil, at no cost.

### 3.3. Adsorption Isotherms

**Figure 7:**
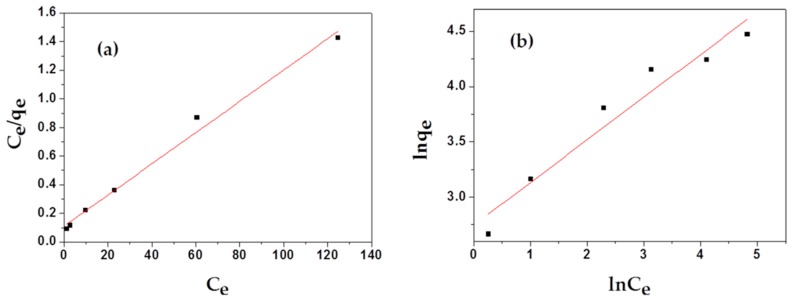
Langmuir (**a**) and Freundlich (**b**) isotherms for the adsorption of MAFC to emulsified oil.

To estimate the adsorption capacities of as-prepared samples, two main isotherm models, Langmuir and Freundlich, were employed to study the adsorption isotherm process of MAFC to emulsified oil, as illustrated in Figure 7. As shown in Table 2, Langmuir isotherm (correlation coefficients R^2^ = 0.98793) correlates better with the actual adsorption process than Freundlich isotherm (correlation coefficients R^2^ = 0.93373). The non-dimensional separation factor R_L_ was 0.0975, in the range of 0 < R_L_ < 1, indicating that the adsorption of MAFC to emulsified oil in the studied concentration range was consistent with the Langmuir adsorption isotherm. In addition, the Freundlich isotherm model constant n was in the range of 1–10, indicating that MAFC was effective in the adsorption of emulsified oil, and the adsorption process was dominated by physical adsorption [35]. Meanwhile, the value of 1/n was 0.386 (1/n < 1), indicating that 38.6% of the active sites had the same energy level, and the adsorption process could be described by the standard Freundlich isotherm. However, the adsorption process might have a multi-layer adsorption mechanism with a small linear correlation coefficient.

### 3.4. Effect of Adsorbent Dose

**Figure 8:**
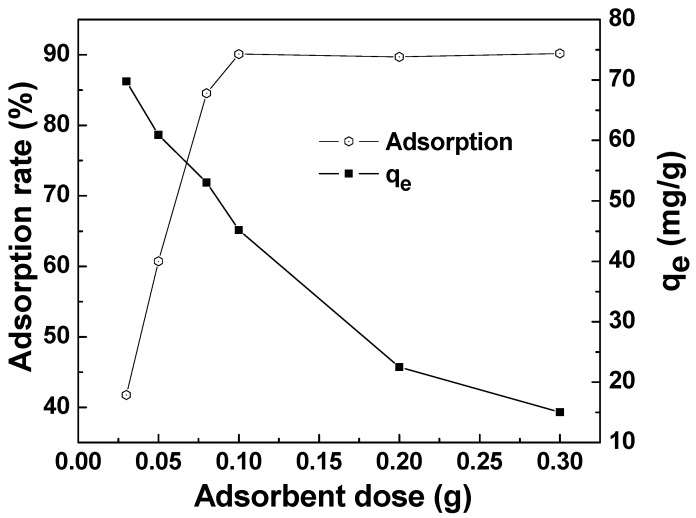
Effect of adsorbent dose on the adsorption of MAFC to emulsified oil (emulsified oil 100 mg/L, contact time 30 min, reaction temperature 25 °C).

The effect of the adsorbent dose on the adsorption of MAFC to emulsified oil is seen in Figure 8. The adsorption capacity gradually decreased with the increase of adsorbent dosage, while the adsorption rate gradually increased and then tended to be stable. This is because more surface active functional groups were available with a greater dose of adsorbent, which could result in a higher adsorption rate. However, the adsorption efficiency was limited by the constant initial emulsified oil concentration. As shown in Figure 8, the adsorption rate of around 90.1% tended to be stable with 0.1 g adsorbent added in 100 mg/L oily wastewater, and the adsorption capacity reduced to 45.05 mg/g. Therefore, we could choose 0.1 g as an optimum dose for MAFC.

### 3.5. Effect of Reaction Temperature

**Figure 9:**
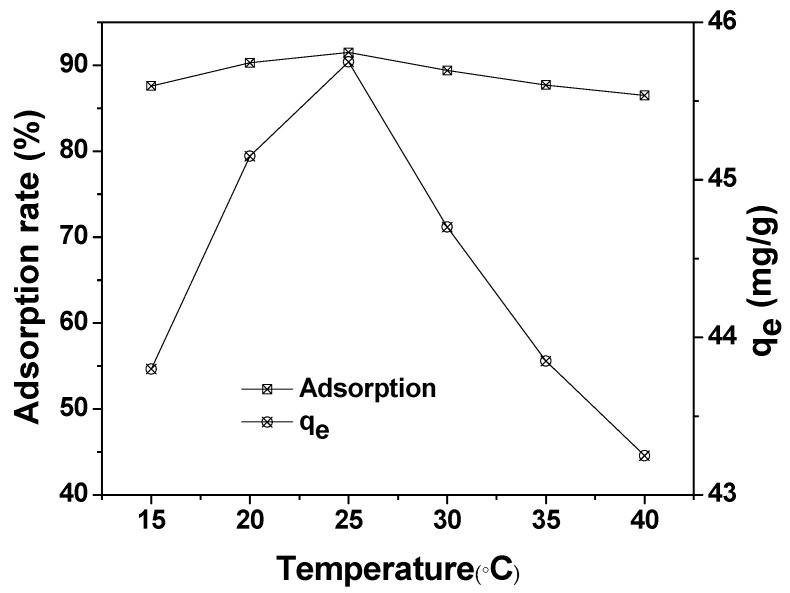
Effect of reaction temperature on the adsorption of MAFC to emulsified oil (emulsified oil 100 mg/L, contact time 30 min, and MAFC 0.1 g).

The effect of reaction temperature on the adsorption of MAFC to emulsified oil (100 mg/L) is illustrated in Figure 9. The maximum adsorption capacity and adsorption rate of MAFC were obtained at 25 °C (45 mg/g). The test data showed that the adsorption efficiency decreased with the increase of the reaction temperature. The increase in the desorption rate of emulsified oil was due to the increase of intramolecular thermal movement speed. In addition, adsorption is an exothermic process, and the increase of the reaction temperature will inhibit the adsorption process. Therefore, it is advisable to choose 25 °C as the optimum adsorption temperature.

### 3.6. Recyclability Studies

**Figure 10:**
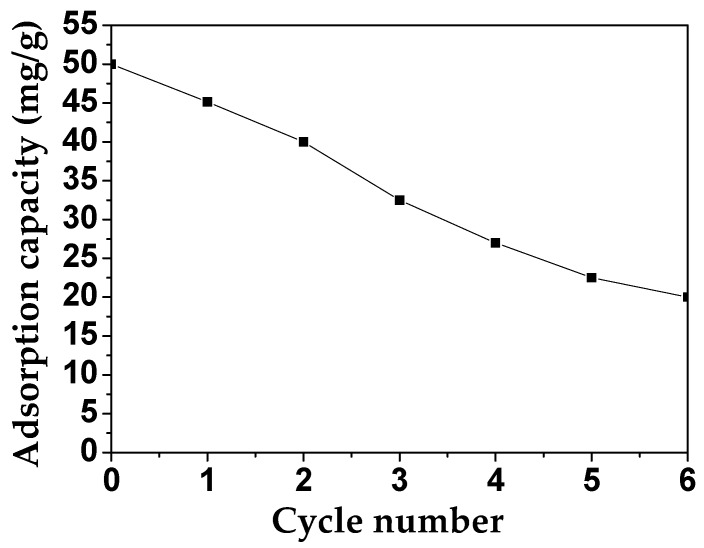
Determination of the recyclability of MAFC based on the adsorption rate of emulsified oil.

For the practical application of MAFC, its recycling performance and its efficiency were estimated. As shown in Figure 10, the adsorption capacity of MAFC to emulsified oil was measured for six testing cycles (each cycle for 60 min). In the first cycle, 45.15 mg/g of emulsified oil was adsorbed, and after all cycles, this adsorption capacity of MAFC still reached 20 mg/g, indicating that MAFC has the stability and efficiency to be used as a good adsorbent.

## 4. Conclusions

A promising charcoal nanomaterial was successfully prepared from fishbone waste. In this work, MAFC exhibited an excellent performance for removing emulsified oil under various experimental conditions. Although the specific surface area of MAFC was relatively small, i.e., 206.0 m^2^/g, compared with other adsorbent materials, its removal rate of emulsified oil could reach 90.1%, which was attributed to the presence of abundant active sites, which were fully exposed after modification by K_2_CO_3_ activation. In addition, excellent HAp crosslinked structures with well-developed porous structures could also increase the adsorption efficiency of MAFC. Therefore, this low-cost, effective, and recyclable charcoal nanomaterial could be a promising bio-adsorbent of organic pollutants in oily wastewater.

## Figures and Tables

**Table 1:** Specific surface area parameters of fishbone charcoal.

Fishbone Materials	BET (m^2^/g)	Total Pore Volumes (cm^3^/g)	Average Pore Diameter (nm)
raw fishbone	0.7	0.003	5.5
FBC	69.8	0.192	10.8
MAFC	206.0	0.3	6.2

**Table 2:** Constants and correlation coefficients of the isotherm and kinetics models.

Adsorption Isotherm					
Langmuir Model			Freundlich Model		
q_max_	b	R^2^	1/n	K_f_	R^2^
100	0.0926	0.98793	0.386	15.55	0.93373
**Kinetics Model**					
**Pseudo-First-Order Kinetics Model**			**Pseudo-Second-Order Kinetics Model**		
q_e_	K_1_	R^2^	q_e_	K_2_	R^2^
11.19	0.04	0.70651	46.08	0.02	0.99996
